# Exploring convolutional neural networks and spatial video for on-the-ground mapping in informal settlements

**DOI:** 10.1186/s12942-021-00259-z

**Published:** 2021-01-25

**Authors:** Jayakrishnan Ajayakumar, Andrew J. Curtis, Vanessa Rouzier, Jean William Pape, Sandra Bempah, Meer Taifur Alam, Md. Mahbubul Alam, Mohammed H. Rashid, Afsar Ali, John Glenn Morris

**Affiliations:** 1 Department of Population and Quantitative Health Sciences, School of Medicine, Case Western Reserve University, Cleveland, OH USA; 2 Les Centres Haitian Group for the Study of Kaposi’s Sarcoma and Opportunistic Infections (GHESKIO), Port-au-Prince, Haiti; 3 Department of Geography, Kent State University, Kent, OH USA; 4 Emerging Pathogens Institute and Department of Medicine, College of Medicine, University of Florida, Gainesville, FL 32601 USA; 5 Emerging Pathogens Institute and Department of Environmental & Global Health, College of Public Health and Health Professions, University of Florida, Gainesville, FL 32601 USA

## Abstract

**Background:**

The health burden in developing world informal settlements often coincides with a lack of spatial data that could be used to guide intervention strategies. Spatial video (SV) has proven to be a useful tool to collect environmental and social data at a granular scale, though the effort required to turn these spatially encoded video frames into maps limits sustainability and scalability. In this paper we explore the use of convolution neural networks (CNN) to solve this problem by automatically identifying disease related environmental risks in a series of SV collected from Haiti. Our objective is to determine the potential of machine learning in health risk mapping for these environments by assessing the challenges faced in adequately training the required classification models.

**Results:**

We show that SV can be a suitable source for automatically identifying and extracting health risk features using machine learning. While well-defined objects such as drains, buckets, tires and animals can be efficiently classified, more amorphous masses such as trash or standing water are difficult to classify. Our results further show that variations in the number of image frames selected, the image resolution, and combinations of these can be used to improve the overall model performance.

**Conclusion:**

Machine learning in combination with spatial video can be used to automatically identify environmental risks associated with common health problems in informal settlements, though there are likely to be variations in the type of data needed for training based on location. Success based on the risk type being identified are also likely to vary geographically. However, we are confident in identifying a series of best practices for data collection, model training and performance in these settings. We also discuss the next step of testing these findings in other environments, and how adding in the simultaneously collected geographic data could be used to create an automatic health risk mapping tool.

## Introduction

Informal settlements remain one of the greatest public health challenges due to the nexus of a variety of disease causing systems (such as extreme poverty, overcrowding, lack of local services and health care), and generally poor data to help guide solutions. While many of these problems might seem unassailable, there are advances that can be made in improving the way that ground level data are collected, processed and utilized by local area public health workers and epidemiologists. In this paper we advance such data acquisition and utilization using machine learning. More specifically we utilize ground-level spatially encoded video and show how environmental risks such as mud and standing water [32] can be automatically as a precursor to near-real time mapping.

Informal settlements should not be considered as homogenous environments as risks vary considerably based on factors such as local elevation, ground type and slope, and local flooding risk. There is also temporal dynamism to these risks, so being able to spatially target prevention or mitigation efforts is vital in fully leveraging limited resources [36]. While different solutions have been utilized to improve on-the-ground spatial detail, such as participatory mapping approaches [16, 35], or through crowd sourcing platforms such as Map Kibera [8], these tend to be cross sectional in nature because of the logistical problems faced during data collection. Indeed, the data deficiencies found in such environments are well documented, and even when on-the-ground technological advances are utilized, meaning solutions designed to collect the required risk data for localized mapping, they tend to lack the sustainability and granularity required for analysis and intervention [19]. Adding further complexity is that these environments are dynamic in nature; the dramatic difference encountered between wet and dry seasons being one obvious example [46]. Further dynamism occurs with critical infrastructure, for example water points (W.Point) or toilets [26], require frequent updating, not only in terms of shifting locations [4, 9, 9, 13, 13] but also on how their quality and risks vary temporally [10].

One such previously employed technological advance used to map health risks, and to provide micro geographic support for more traditional epidemiological surveillance in these environments, is the spatial video (SV) [11, 12]. This field technology consists of a global positioning system (GPS) enhanced video, which for informal settlements is usually hand carried through the study space [43]. Each resulting video frame has an associated GPS coordinate attached, meaning the media becomes a digitizing source [10], with water points, drains, standing water, mud, and even trash being mapped [4]. Conceptually, this approach can support local public health operations, or just serve as a basic mapping tool for the local populace [17]. Yet while it has proven successful in limited operation, the previously identified problem of scalability and sustainability still remain.

Yet this method and these data are worth further exploration to investigate how they can be made more useful to local stakeholders. As an example of previous SV use, monthly water samples were taken to assess localized bacterial risk in Port-au-Prince, Haiti. This epidemiological study which resulted in spatio-temporal mapping of water “risk”, also included concurrent SV surveys to record the associated environment [9–11, 13]. These additional visual records provided alternative explanations for local temporal variations in fecal coliform counts which otherwise would have been assumed to be caused by environmental or meteorological factors. For example, the SV captured the decaying nature of the concrete around a well, or the amount of standing water where people would rest their water buckets, both of which can contaminate the water. While the water samples provided vital biological insights into health risk the SV contextualizes those locations with details that can explain causations and patterns. However, the mapping process involves a labor intensive viewing of the video and then digitizing risks into a geographic information system (GIS) layer. If SV were to be used as a more sustainable method for map creating and updating, a two-step process of automatically identifying the risk features and then mapping them is quintessential. In other words, reducing the human effort involved. In this paper we consider the first step in the process, automated risk feature extraction using machine learning and identifying the specific complexities associated with data collected from these environments.

Recent developments in the area of machine learning, especially due to the revival of deep neural networks, offers opportunities to tackle challenges such as image classification [49], object detection [41], semantic segmentation [31], speech recognition [24], machine translation [3], and natural language processing [23] With the development of a particular class of deep neural networks called convolution neural networks (CNN) [29], considerable progress has been made in image classification, object detection, and semantic segmentation. Compared to traditional fully connected multilayer perceptron architectures where every neuron is connected to every other neuron, CNN supports weight sharing where a neuron is connected only to the neurons that are within its receptive field. Along with being highly memory efficient, this type of architecture can capture fine scale spatial and temporal dependencies when compared to fully connected architectures. This property of CNN makes it particularly attractive for tasks involving both 1 dimensional (for example time series data), and 2 dimensional gridded data (image data). The key to success of CNN or any other deep neural network architecture is the availability of large training datasets (which helps in better generalization), and high performance computational resources. While the availability of high performance computational resources continues to improve (especially owing to the development of GPU (Graphical Processing Unit) and TPU (Tensor Processing Unit) based architectures), the availability of large training datasets is always a domain specific challenge. As previously stated, not only do informal settlements pose considerable health problems, but they are also notoriously data poor, meaning that there is scant training data. The use of remotely sensed imagery as a data source to utilize machine learning including CNN has been tried for various health risks prevalent in informal settlements all around the world [1,18, 25, 30, 44, 48, 50]. Of more relevance to this project, at least in terms of the data source if not the same environment, is the analysis of high resolution “neighborhood” imagery from sources such as Google Street View (GSV). For example, Rad et al. [37], in their work on localizing and classifying waste on the streets, used an acquisition system mounted on a vehicle to collect street images which were then input for a deep CNN to identify litter and trash. Chow et al. [6], utilized deep CNN on GSV to evaluate built environment characteristics such as building density, aesthetics, disorder, pedestrian safety, and bicycle infrastructure. Mooney et al. [33] also extracted physical disorder from GSV images, while Law and colleagues [28] developed Street-Frontage-Net to evaluate the quality of street frontage for signs. The gap, therefore, is that the type of data available for informal settlements is not of the type found to be most useful for identifying street-level risks such as trash, standing water and water points.

There is no easy solution to solve this gap; online visual data suitable for automated image classification in informal settlements is scarce, especially when the additional problem of how these environments change geographically; similar settlements in Haiti and Ghana have similar problems and features, but the details needed for image classification vary considerably. While remotely sensed imagery can be improved with other data sources [18] such as local censuses, there is still a need to contextualize local environment at the street scale [45] with on-the-ground imagery to improve the generalization and accuracy of machine learning models. While normally collecting these types of data are logistically challenging, the project team for this study has been using SV in multiple environments and time periods, amassing a considerable library of granular environmental imagery which can be used to explore various aspects of model training for these types of settings.

Not only does such a library allow for experimentation with and improvement in image classification, but also input considerations can also be quantified, such as how localized challenges in field data collection affect input data quality and prediction. This is important as it is not realistic to think that there is a set of clean images readily available for any environment, and where collection occurs (for example in tight urban corridors or within drainage channels), the perceived safety of the data collector, and variations in camera make and type can all lead to issues such as angle of view, considerable movement within frame and general image quality. If SV is to become more sustainable translational method for local mapping, it is important to see how much of a problem these variants cause for the predictive model. This paper addresses these points by using the SV archive to examine the effectiveness of machine learning *on ground level imagery*, for multiple informal settlements in Haiti. Our results, which are focused on identifying environmental health risks, are a first step towards using automatic risk detection as part of a real-time mapping tool.

## Methods

With the recent advancements in CNN, various new object detection algorithms have emerged including R-CNN [22], Fast R-CNN [21], Faster R-CNN [42], and YOLO (You Only Look Once) and its variants [37–38]. R-CNN and its variants uses a two-step process for object detection. In the first step, interesting parts of the image are selected through a Regional Proposal Network (RPN) technique, and in the second step a CNN is used to classify an object from the regions selected by RPN. Compared to the two-step process of R-CNN, the YOLO method [39], unifies the target localization and object detection as a single regression problem. A single neural network predicts the bounding boxes and class probabilities for all the objects. As it’s a single step process with the algorithm traversing through the image only once, YOLO is much faster when compared to R-CNN and its variants. Subsequent versions of YOLO (YOLOv2 [38] and YOLOv3 [40]) improved the method, having more convolution layers, has better accuracy and efficiency. For this study we have used YOLOv3 as the object detection algorithm.

### YOLOv3 architecture

**Fig. 1:**
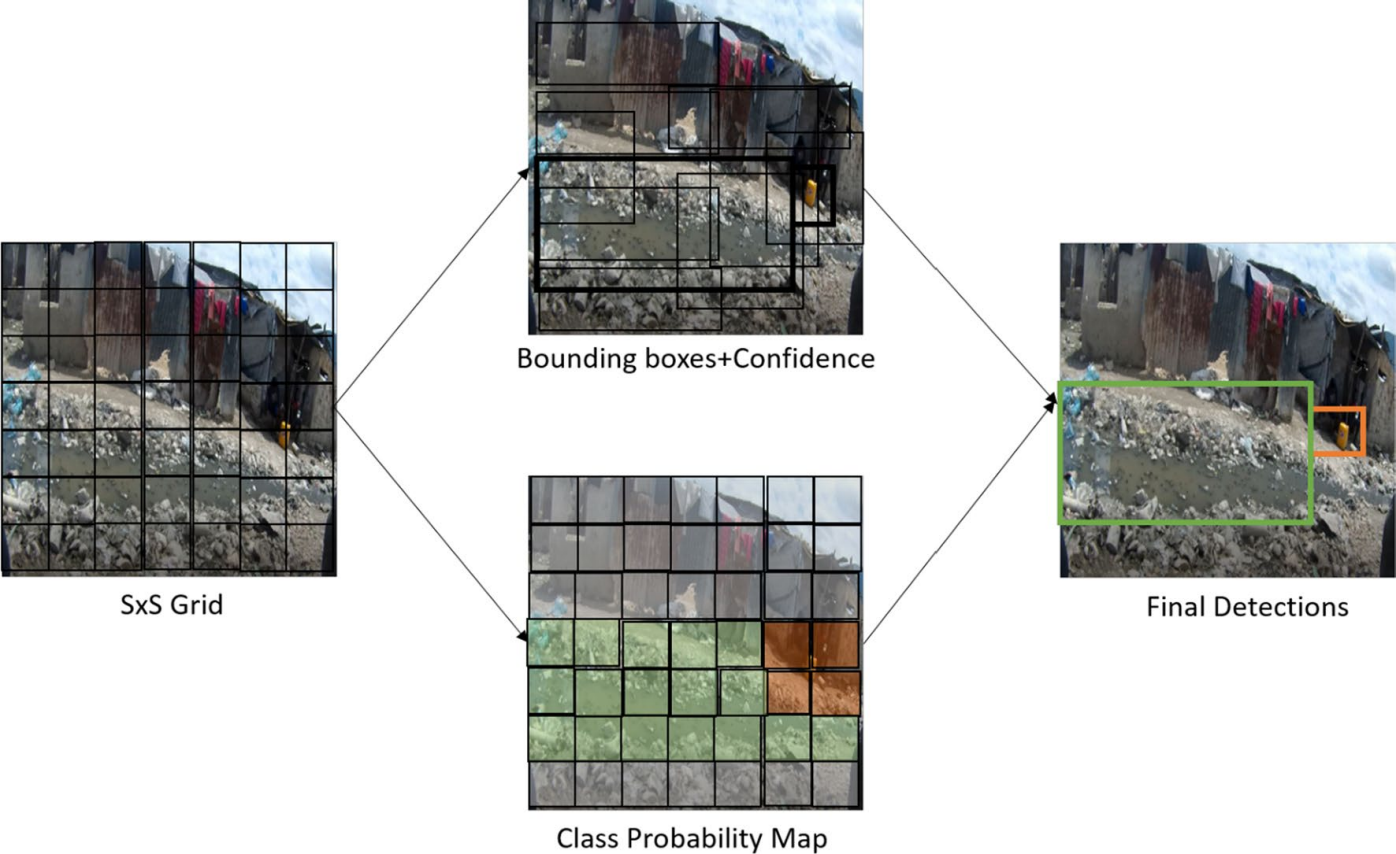
The YOLOv3 model. Object detection is posed as a regression problem

YOLOv3 utilizes Darknet-53 [40] as its backbone network for feature extraction. Each image in the training set, for example the muddy water (M.Water) seen in Fig. 1, is divided into a 2D matrix of NxN (N usually 7) grid. The network outputs five bounding boxes for each grid cell along with an “objectness” score for each bounding box. It also outputs K class probabilities where K represents the total number of classes. Thus each grid produces a total number of 25 + K (5 × 4 + 5 + K) values. Rather than predicting the absolute coordinates of the bounding box centers, YOLOv3 predicts an offset relative to the coordinates of the grid cell. For each grid cell, YOLOv3 is trained to predict only the bounding boxes whose center lies in that grid cell. Confidence for predictions in each of the grid cell is given by Eq. 1.

1 $$Confidence={p}_{r}\left(Object\right)* {IOU}_{pred}^{truth}, {p}_{r}\left(Object\right)\in \{\mathrm{0,1}\}$$

Here p_r_(Object) is 1 if the target is in the grid and 0 otherwise. ${IOU}_{pred}^{truth}$(intersection over union) is defined as the overlap ratio between the predicted bounding box and the true bounding box (Eq. 2). The confidence provides estimates about whether a grid contains an object and the accuracy of the bounding box that the network has predicted.

2 $$IoU=\frac{{S}_{overlap}}{{S}_{union}}$$

In-order to reduce the detection error, anchor boxes which are a priori bounding boxes (5 for each grid), are generated by using a k-means algorithm applied to the height and width of the training set of bounding boxes. These make the network more likely to predict appropriate sized bounding boxes which also speeds up training [40]. For training, YOLOv3 uses sum-squared error in the output as the optimization procedure. The loss function is a combination of errors on the bounding box prediction, object prediction, and class prediction (Eq. 3).

3 $$Total Loss= {Error}_{coord}+{Error}_{iou}+{Error}_{cls}$$

### Generating training images for YOLOv3

**Fig. 2:**
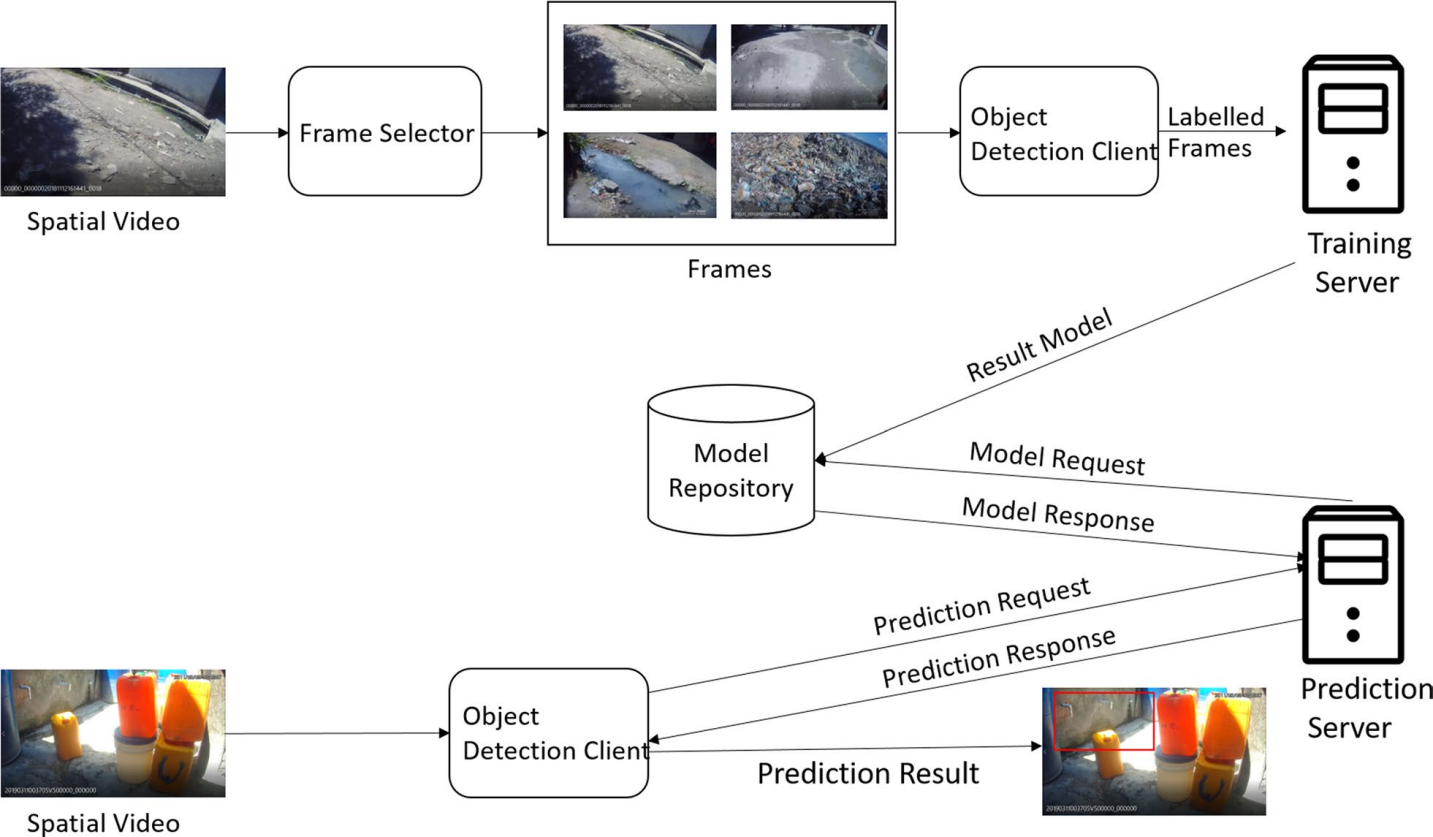
Schematic flow diagram for object detection pipeline. The top section indicates training workflow while the bottom section indicates prediction workflow

The schematic flow diagram for the entire SV object detection pipeline is shown in Fig. 2. In order to address the problem of varying image quality on model training a bespoke standalone software (Frame Selector) was developed to mine the SV image archive. This software facilitated user selected images to be extracted as single frames for each of the environmental category types. As each frame is associated with a particular time, that same time can also be used to extract the corresponding frame from the source video. The software can be downloaded from https://cwru.box.com/s/iz8nl1ijqwzpr1094b66rivkllg9249j

### Image labelling and training

**Fig. 3:**
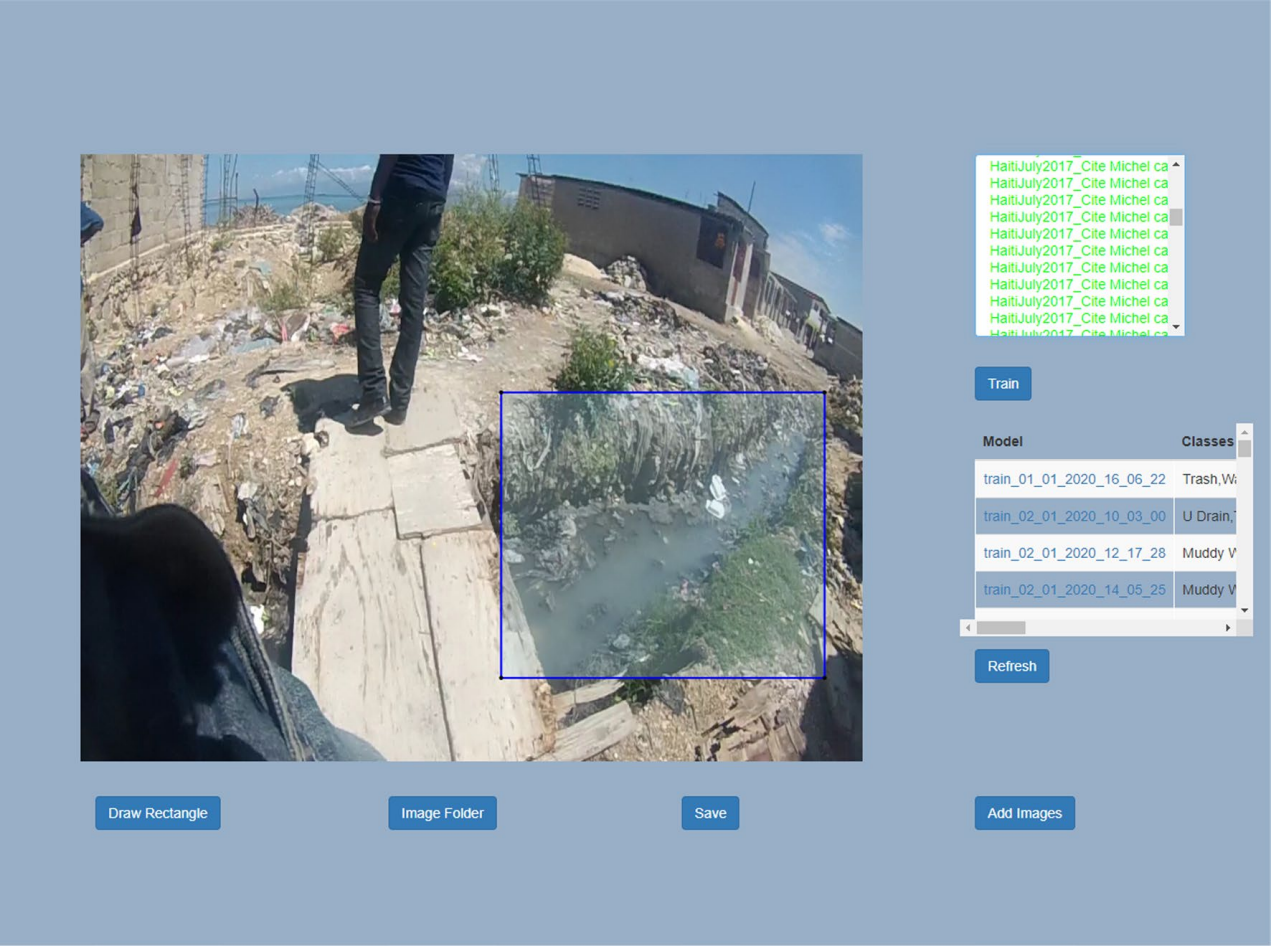
Object Detection Client software for labelling training images. A rectangular bounding box is drawn around a risk feature, in this example a drain, and the object is then labelled from a set of drop down options. The table on the right represents the models that are already trained

The frames extracted using the Frame Selector software is further labelled using the Object Detection Client software (Fig. 3). Each extracted frame is labelled with five values including the center coordinates (x, y), the width (w) and height (h) of the bounding box for the object (normalized to a value between 0 and 1), and the class to which the object belongs. The details of all the images, its labels and bounding box dimensions, are stored as a JavaScript Object Notation (JSON) file for further retrieval and processing.

A frame “packet” containing all the image frames, all corresponding labels, separate text files indicating the images used for training and the images used for validation, and a configuration file are generated once the labelling process is finished. The frame “packet” is sent to the training server for processing and the resulting model file is saved in a common repository.

### Prediction

**Fig. 4:**
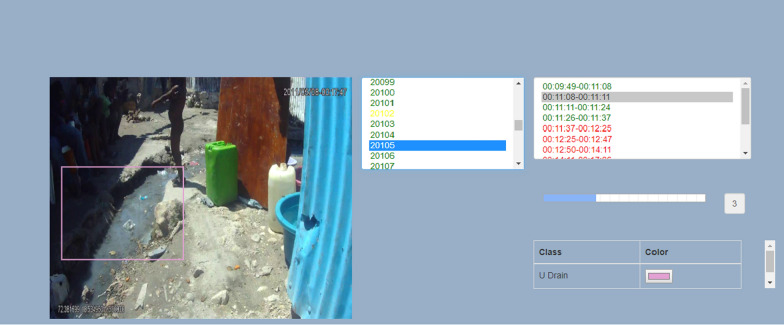
Prediction screen for the software. The frame window displays the image along with the results. The rectangular box represents the predicted bounding box. The timestamp selection dropdown indicates the associated time sections in the video and the frame selection dropdown indicates the corresponding frames in the video

For prediction and inference, a video file is converted into packets of images based on the frequency parameter set by the user. As an example, if the selected frequency parameter is 5 then every 5th frame of the video is stacked together to form packets of 20 images. The frequency parameter can act as a trade-off between the image processing time and the overall detection accuracy. A low frequency parameter would select more image frames which in-turn increases the processing time though improving the detection accuracy. Each packet, along with the information about the trained model are sent to the prediction server as a POST request in an asynchronous fashion. The prediction server loads the training model and runs inference on the image packets to generate a single JSON file containing the predictions for each frame. The prediction results include the center (x, y) of the object detection box, its width and height along with the class and the probability of the object being successfully labelled. On receiving the results from the prediction server, the image frame along with the object prediction as rectangular box are displayed (Fig. 4).

## Data and experimental setup

Beginning in October 2016, monthly water samples were collected from public water points drainage channels or the coast-land interface in multiple informal settlements of Port-au-Prince, Haiti [9, 13]. Along with the water samples, the field team also collected micro environmental surveys using SV [9, 13], primarily of the water sample locations but also along the paths connecting them. These routes contained multiple examples of environmental factors commonly associated with different disease risks,muddy/standing water (for example dysentery, open drains (for example cholera) trash (for example malaria), and animals (for example leptospirosis). After approximately three years of data collection, the resulting SV archive provided an excellent resource to extract images representative of health risk features for this area, in category types suitable for model training [2, 5, 20]. In addition, the category “animal” utilized a pre-existing model trained on OpenImages [27] for prediction with no additional training. To summarize, example images for each of the environmental risk categories were extracted and used to train the model for that feature. A second set of videos were then manually classified for the presence of those same types of environmental risks. These were then used to check the accuracy of the predictive model.

The second set of 12SVs used to assess the accuracy of the different models were chosen to evaluate different types of neighborhoods/environments, different time periods for the same neighborhood, and variation in image type and quality. These included coastal reclaimed land (A, C and D), variations in road and building type including paved densely packed streets (B), and varying elevations (E).^1^ The majority of the SVs were collected while walking with either an extreme sports camera (a Contour Plus 2), or body cameras (MIUFLY or Patrol Eyes). The quality of the video was generally good (1280px), though the camera angle varied from being pointed downward as the person walked the route, to being aimed specifically at a feature being captured such as a water point. As a result, the video angle also varied resulting in a set of non-standard training images that would, probably, be rejected for most developed world projects.

For the first round of testing, image resolution was set at 608px (same as the training resolution), and the frame frequency parameter was set to 10 frames (every 10 frames would be used for prediction). After the prediction, every packet of video frames containing 100 images were analyzed for positive matches in concordance with the datasheet of risk matches for the video. Therefore, if a water point is labelled at time 12 min and 45 s, the corresponding video frame is analyzed for a match (Fig. 4).

A frame frequency parameter was introduced to extract frames from the video at different intervals to reduce processing time. In order to understand the impact this skipping of image frames might have on object detection, we performed a second round of testing with a frame window concept, where all the frames that fall within an interval window are selected for object detection. As an example, if there is an object occurring at 12 min and 45 s in an image frame and the frame window size is set to 60 frames, then all 30 frames behind and ahead of the timestamp are used for object detection. In-order to extract the relevant frames, the timestamp is converted to a frame number by multiplying the frame rate (number of frames per second) with the video time in seconds. Unlike with the first round, only frames that have a potentially matching object are checked for a positive match.

To further understand the impact of image resolution on prediction, a third round of testing was conducted with varying image resolutions including 224px, 416px, 832px, 1024px, and 1280px (most of the original images are at this resolution). Those images that were unclassified for the first two rounds (with 608px) were only used in the third round. Along with the prediction results, other performance measures such as total program runtime, variation in performance with frame stack size and variation of run time and memory utilization with changes in image resolution were also noted.

## Results

**Fig. 5:**
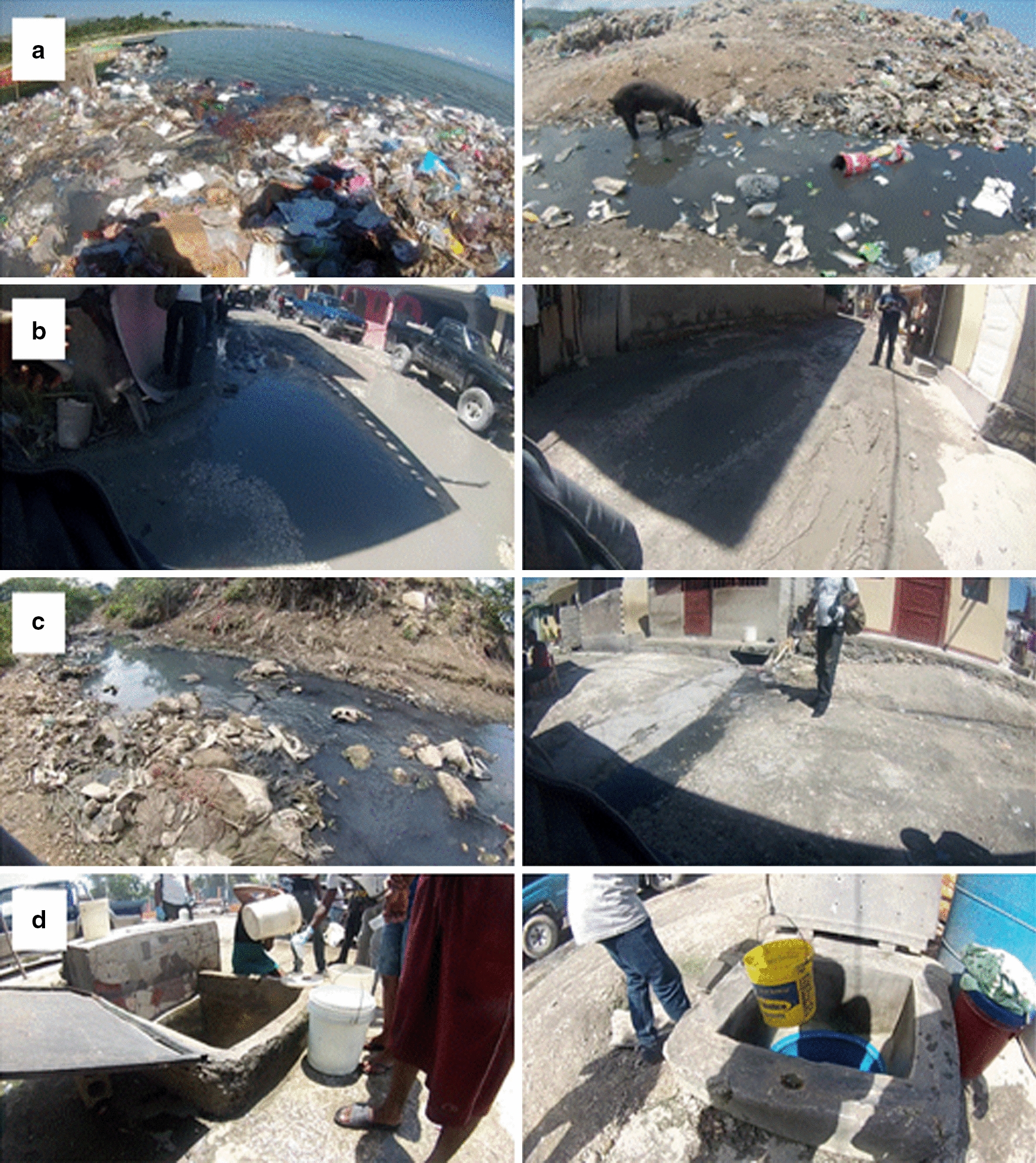
Example training images used for labelling **a** Trash, **b** Muddy Water, **c** Drain, and **d** Water Point

The total number of images and objects for each category is shown in Table 1. The training to validation ratio was set to 10:1 as the number of images was still low for an object detection task (generally ranges well above 1000 images for a single class), and the split was done randomly. While the images in Fig. 5 are examples utilized for training, it was found that there was considerable variation within each category, both in terms of image type and size, including overlaps between categories. For example, Fig. 5a, b could both be labelled as a drain. The images in Fig. 5c are two different size drains, though not shown here are the engineered drains with distinct concrete sides, and more naturally occurring channels which might also be categorized as a stream. Water points (Fig. 5d) varied in type, so much so that a second round of image extraction was needed to include more taps and the (usually white) pipes connected to them. This “fuzziness” is typical for informal settlements where the whole environment tends to be unplanned and rather haphazard leading to a lack of image clarity found in most similar developed world projects.

**Table 1:** Details of images used for training. For water point an additional set was added due to a lack of images containing pipes and taps

Category	Total images	Total objects	Total images(II)	Total objects(II)
Drain	98	98	–	–
Trash	67	84	–	–
M.Water	74	86	–	–
W.Bucket	49	96	–	–
Tire	55	88	–	–
W.Point	59	61	94	104

The training hyper-parameters and other details such as image resolution are shown in Table 2.^2^

**Table 2:** Initialization parameters for the training algorithm

Image Size	Mini-batch Size	Total Epochs	momentum (SGD)	Initial Lr	Final Lr	Weight Decay
608px	1	273	0.97	0.002	-0.04	0.0004

*Lr* learning rate

**Fig. 6:**
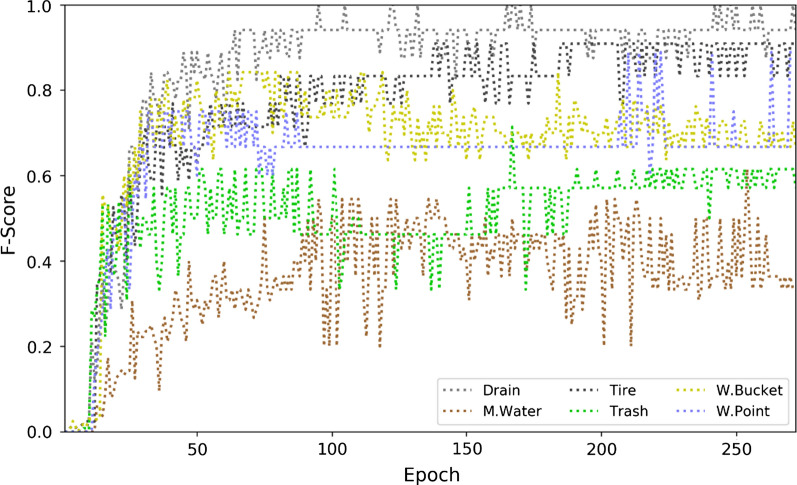
F-Score vs Epoch for the six different health risk categories

A common metric used to indicate the quality of a classification model is the F-score which is essentially the harmonic mean of the precision and recall. The model trained for identifying drains had almost a perfect F-score (around 1) after complete training (273 epochs), while the model trained for muddy water had the lowest F-score (around 0.4) (Fig. 6). The F-score for trash was around 0.5, for water points averaged between 0.6 and 0.7, for water buckets was between 0.7 and 0.8, and for tires was 0.8 to 0.9 (Fig. 6).

**Fig. 7:**
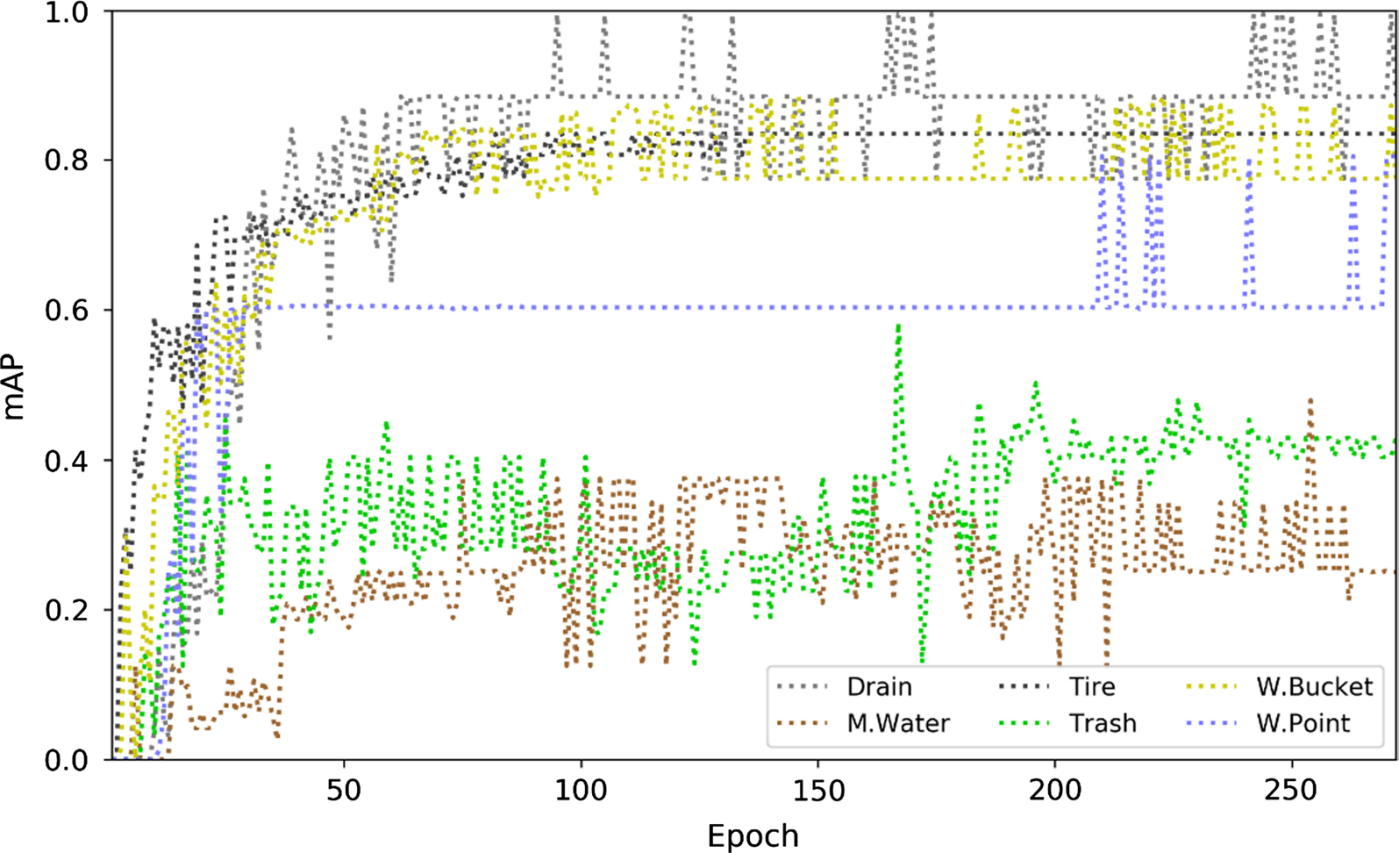
Mean average precision (mAP) vs Epoch for the six different health risk categories

While the F-score helps to gain insight into the classification accuracy of the model, the mean average precision (mAP) provides a better understanding of detection by considering the Intersection over Union (IoU) (Eq. 2) criteria. IoU determines whether the bounding box for the objects was also correctly predicted. In order to assign a prediction as a “match”, the label has to be correct and the IoU should be above a certain threshold (normally 0.5). The mAP vs epoch graph for all the six categories show that the muddy water and trash classifiers had a low mAP (20% to 40%), while the classifier for water point had a mAP around 60%, and classifiers for water buckets (mAP around 75%), tires (mAP around 80%), and drain (mAP around 85%) had relatively high mAP (Fig. 7).

**Fig. 8:**
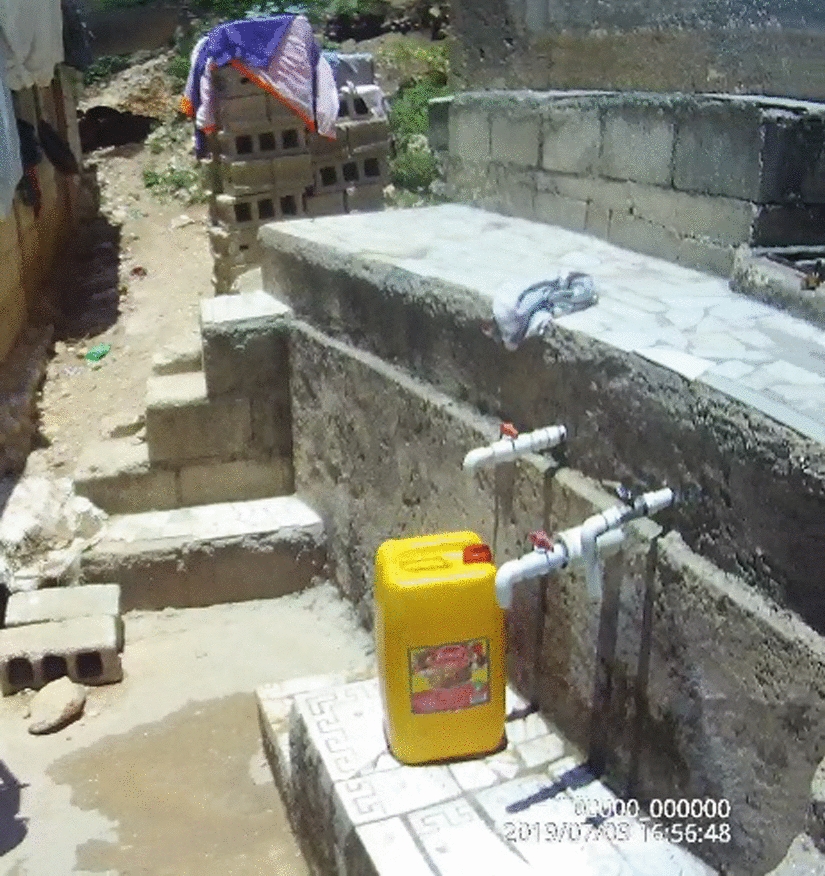
Positive object detections after 1^st^ round of accuracy testing. The resolution for the image was set at 608px. Frame frequency was set to 10 frames. Positive object detection examples for **a** Muddy Water, **b** Drain, **c** Trash, **d** Water Buckets, **e** Tire, **f** Animal, and **f** Water Point

**Fig. 9:**
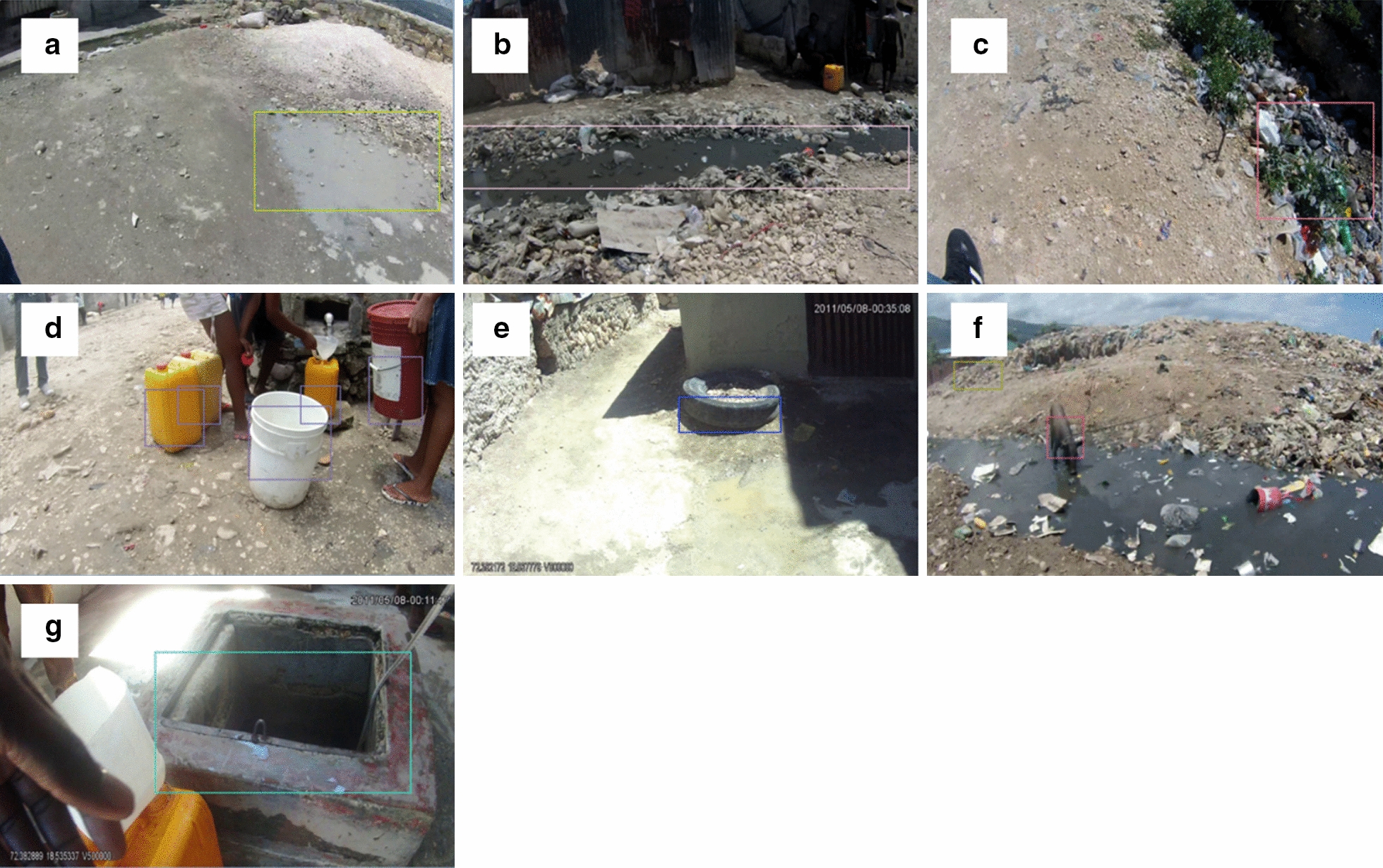
Positive object detections after 2nd round of accuracy testing. The resolution for the image was set at 608px. A frame window of 60 was selected for detection. Positive object detection examples for **a** Muddy Water, **b** Drain, **c** Trash, **d** Water Buckets, **e** Tire, and **f** Animal

For the first round accuracy testing with an image resolution of 608px (Fig. 8) and a frame frequency of 10, classifiers for drain and water buckets had the highest accuracy at 91% and 95% respectively (Table 3). Classifiers for tires and animals had a medium level of accuracy at 86% and 82% respectively, while classifiers for water point, trash and muddy water performed worst with an accuracy of 73%, 72%, and 68% respectively (Table 3). For the second round of accuracy testing with a frame window of 60 frames, every classifier except for water points increased in accuracy (Fig. 9). The accuracy of the classifier for drain increased from 91 to 97%, while the accuracy for the water bucket classifier increased from 95 to 97% (Table 3). Classifiers for tires and animals which had medium accuracy in the first round (86% and 82%) rose to 91% (Table 3). Of the initially poorest performing classifiers, trash and muddy water, accuracy improved to16.6% and 20.5% respectively. By analyzing the image results for water points, we found that the initial training dataset lacked a suitable breadth of images, especially single pipe based water points which led to the poor predictions. To tackle this issue, we added an additional set of 35 images from the SV archive with pipe-based water points (Fig. 8). After adding the new set of images the accuracy for the water point classifier increased by about 15% (Table 3). Finally, by changing the detection resolution at various levels (from 224 to 1280px), the accuracy for models trained to classify drains (2% increase), trash (14.2% increase), muddy water (14.6% increase), and water points (9.5% increase) all improved, while the remaining models had no change in classification accuracy (Table 3).

**Table 3:** Prediction accuracy test results for drain, trash, muddy water (M.Water), water point (W.Point), water bucket (W.Bucket), tires, and animals

Video#	Drain				Trash				M.Water							
	T	R1%	R2%	R3%	T	R1%	R2%	R3%	T	R1%	R2%	R3%				
1	15	100	100	100	10	100	100	100	9	78	89	89				
2	20	95	95	100	19	74	84	100	62	65	77	92				
3	13	100	100	100	25	76	84	100	38	74	89	95				
4	15	100	100	100	11	64	73	100	6	83	83	100				
5	6	50	100	100	15	27	53	93	8	38	38	75				
6	7	86	86	100	40	75	85	93	65	83	95	100				
7	21	100	100	100	21	57	86	90	31	55	81	97				
8	6	100	100	100	7	100	100	100	5	80	100	100				
9	4	75	75	75	16	88	94	100	11	45	73	100				
10	5	80	100	100	43	72	84	98	31	55	71	87				
11	10	100	100	100	17	76	88	94	13	69	85	100				
12	13	62	92	92	15	80	80	93	29	72	72	90				
Totals	135	91	97	99	239	72	84	96	308	68	82	94				

W. Point					W. Bucket				Tire				Animal			
T	R1%	R2%	R3%	R4%	T	R1%	R2%	R3%	T	R1%	R2%	R3%	T	R1%	R2%	R3%
4	100	100	100	100	16	88	100	100	1	100	100	100	5	100	100	100
6	50	50	83	83	27	96	96	96	18	83	89	89	7	86	100	100
3	100	100	100	100	11	100	100	100	2	100	100	100	3	67	67	67
7	86	86	86	86	9	100	100	100	1	0	100	100	1	100	100	100
5	60	60	60	80	9	78	78	78	14	93	93	93	7	86	100	100
3	33	33	67	67	4	100	100	100	2	50	50	50	8	100	100	100
4	25	25	100	100	9	100	100	100	25	88	92	92	6	67	83	83
1	100	100	100	100	1	100	100	100	1	0	0	0	1	0	0	0
3	33	33	33	100	2	100	100	100	4	100	100	100	2	100	100	100
5	80	80	80	100	2	100	100	100	3	100	100	100	3	67	100	100
2	100	100	100	100	3	100	100	100					1	0	0	0
8	100	100	100	100	12	100	100	100	7	86	100	100				
51	73	73	84	92	105	95	97	97	71	86	91	91	44	82	91	91

Runtime statistics (Table 4) for the accuracy test indicate that prediction time for a single image frame is almost the same for all image resolutions. This is important as the total number of frames that can be stacked together to form a single packet for running predictions varies with image resolution. Images at lower resolutions (224px or 416px)

**Table 4:** Runtime statistics for predictions at various resolutions

Resolution	Prediction time for single frame (s)	Max stack size	Prediction time for max stack (s)
416px	0.03	70	0.03
608px	0.02	30	0.02
832px	0.02	15	0.02
1024px	0.02	10	0.02
1280px	0.02	5	0.02

consume less memory and can be efficiently processed by stacking up a large number of frames to form packets.

## Discussion

Informal settlements are a challenging mix of different health challenges and poor available data. Previous use of machine learning classification for these environments have utilized overhead remotely sensed imagery to identify and map their geographic extent. While successful at this relatively coarse spatial scale, for public health intervention there is a need for street and house level data. Only at this scale, with these types of data, can an accurate assessment of the interaction between living conditions and potential environmental health risks be identified. To use machine learning at this scale, to capture factors that often occur beneath the overlapping building canopy and therefore beyond normal remotely sensed imagery [45], a new image library is required. These data also need to be longitudinal given the dynamic nature of these spaces, with significant changes occurring at different cadences, both seasonally and then from year to year [9, 13]. To be able to create a sustainable way to identify and map health risks could prove vital for health intervention initiatives. Unfortunately, when cross-sectional mapping efforts are mobilized, benefits are limited as there is little chance of repeat data collection and mapping due to resource limitations. One possible solution is SV, a data collection method that has been successfully used in informal settlements in various countries. The method itself is relatively easy to use. The challenge is in how to turn these data into knowledge in the form of local maps. The first step addressed in this paper is using machine learning to effectively classify these video archives into objects labeled as being a health risk.

An ongoing project in Haiti supporting local epidemiological investigations has produced a SV archive to both train a series of machine learning models and then test their resulting ability to identify environmental risk factors. Model output shows that this is indeed a viable approach to classifying environmental risks. The model performance output, as seen in the F-Score (Fig. 6) and mAP (Fig. 7) graphs reveal that this approach works best for “distinct” objects such as drains, tires, and buckets, though there is less success in identifying more “fuzzy” features such as trash and muddy water. From a training perspective this is because these objects have a well-defined structure (edges and corners) and can be more easily “learned”, while trash and muddy water are often more amorphous and as such pose a greater challenge for the learning algorithm to extract the relevant features. From a health perspective this means that some features with known health risks, such as tires [14, 34] (mosquitos) or drains [15] (enteric disease or drinking water contamination) can even now be easily identified for mapping purposes. Of more concern is the ability to correctly identify muddy areas which have been linked to variety of diseases, especially where children play, and trash accumulations where containers provide breeding grounds for mosquitos, attract animals and become dumping grounds for human feces [7]. However, nuances in image detection for these features also revealed possible model improvements. For example, detection success depends on the *scale* or aggregation of the features. For example, a single piece of trash can be more easily identified because of its distinctness but when the volume of trash increases (as does the associated health risk), the mass now including a mix of objects such as bottles, disposable food containers, and plastic covers than a more continuous “trash space” occurs. While still being trash, this aggregation leads to a fuzziness that reduces successful identification. Unfortunately, from a health perspective it is our experience that these types of trash agglomerations are commonplace in informal settlements. Therefore, potentially, the training images selected might need to be reconsidered into subcategories based on an agglomeration to distinctness continuum.

**Fig. 10:**
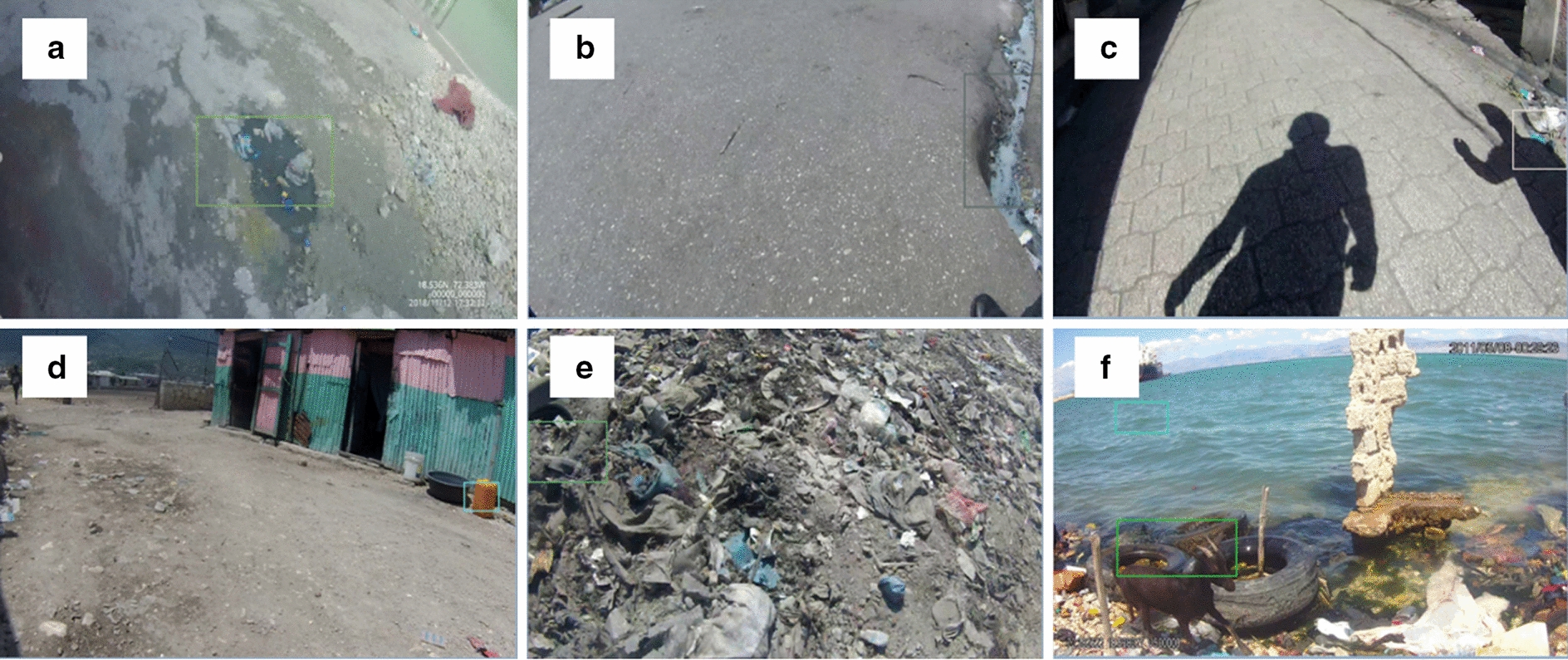
Example of a pipe-based water point that was added to the training set after the second round

Our results also revealed that while some models might be successfully transferable to other sites and even countries (tires being the best example), there is also a degree of location specificity that will be needed for local training. For example, water point detection didn’t improve by increasing the frame rate (no change) or the image resolution (minor change). This was because the initial selection of training images was not broad enough to account for more local variations in water access and we did not initially include enough tap and pipe examples (Fig. 5d). There was a considerable performance gain (R3% for water point) (Table 3) after adding a new set of sample images with taps and pipes to the training set (Fig. 10). It is likely that this type of localized nuance will always be needed in model training. Other potential examples of in situ training might include public toilets, food vendors and health communication signage.

There was a substantial performance improvement in-terms of detection accuracy when more frames were added into the model (R2%) (Table 3). One explanation for this is because of the way data tend to be collected in these environments. A hand held (often semi concealed) camera tends to be used because of the narrow passages and area insecurity. This will often result in considerable variation in image quality, angle and point of focus. For Haiti, while the SV was focused on key features connected to the project, such as water points, which would mean frame sampling is appropriate, on the walking path sometimes only 1 in 10 frames might be suitable for model prediction. Therefore, any form of frame sampling is likely to decrease overall model performance. Having multiple frame options also increases the chance of the feature being identified to be located at the center of the image rather than in the periphery, which again aids in detection. The trade off, of course, is a considerable increase in computation time (Table 4). However, we believe this is an acceptable cost in order to fully leverage the varying quality in the SV.

**Fig. 11:**
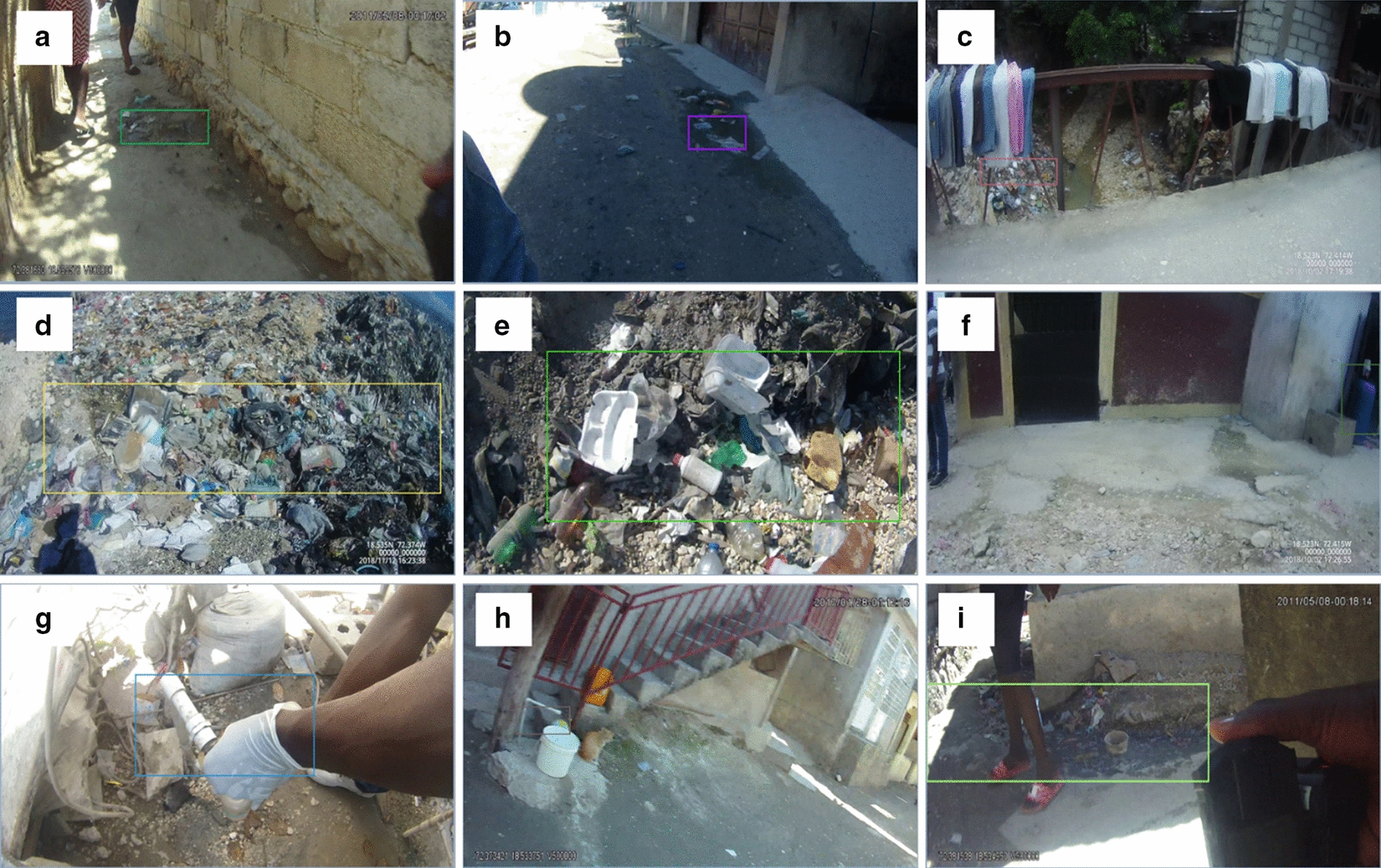
Positive object detections after 3rd round of accuracy testing. The resolution for the image varied from 224 to 1280px. A frame window of 60 was selected for detection. Positive object detection examples for **a** Muddy Water (832px), **b** Muddy Water (1280px), **c** Trash (1280px), **d** Trash (416px), **e** Trash (416px), **f** Water Point (1280px), **g** Water Point (416px), **h** Water Point (1024px), and **i** Drain (416px)

An interesting finding regarding image resolution and testing accuracy occurred in the third round of testing (R3%) (Table 3). Running the detection algorithm at higher resolutions (> 832px), helped to identify objects further from the primary camera focus. As an example, a trash pile that was under a bridge (at a greater focal distance) (Fig. 11c) was only captured by the detection algorithm when the image resolution was set to (1280px). On the contrary, running the detection algorithm at low resolution (224 or 416px) are particularly useful when the object is closer to the camera (Fig. 11e, g). Therefore, for informal settlements there might be a need to use flexible imagery inputs (downscaling when necessary) for certain risks, especially the fuzzier categories such as trash, mud or standing water.

We also experimented with the success of image detection based on the type of video input. SV was selected for different time periods at the same location (to assess stability in detection across time), and different camera models and angles of view. While all these variants cannot be described in this paper, overall the results were encouraging across all camera types. This is important as there is likely to be little consistency in camera types used in different locations, for example recently collaborators have started to use smart phones. Of more importance, as already mentioned, was making sure the camera was pointed at the feature of interest rather than capturing it in the image periphery. For future SV data collections, informing the field team to pay attention to a list of pre-defined risk features would certainly improve model performance. However, even with a more focused intent, there is still the possibility for secondary feature detection, especially if these data are repurposed for other informal settlement and research needs and perspectives. This is an important health consideration, for example, during the current Covid-19 situation, how might these video be used to either identify potential risk areas, or alternatively where testing or vaccination initiatives be targeted.

**Fig. 12:**
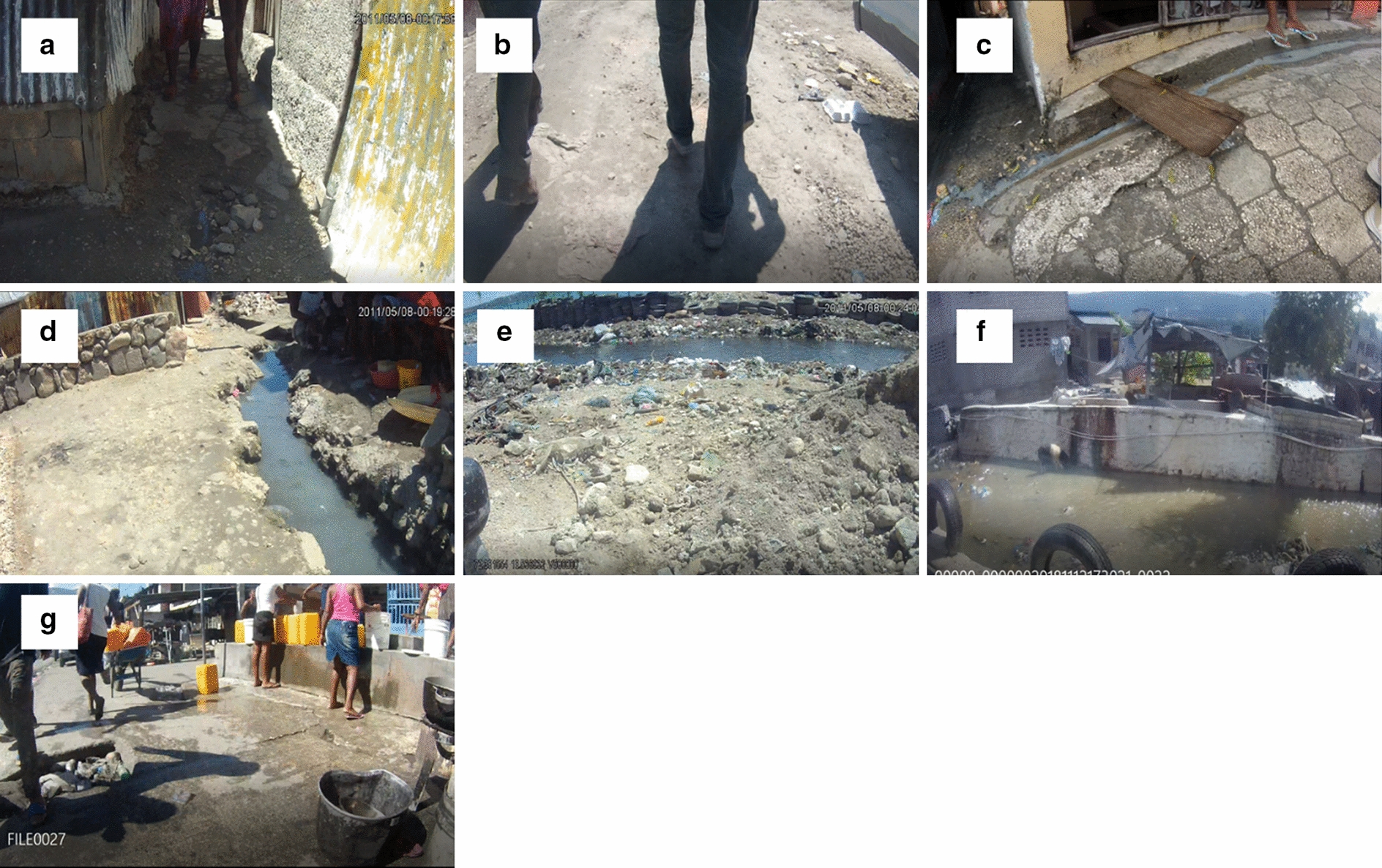
Missed objects after 3 rounds of accuracy testing. **a** Muddy Water, **b** Trash, **c** Drain, **d** Water Bucket, **e** Tire, **f** Animal, **g** Water Point

To improve the detection accuracy for fuzzy risks such as trash and muddy water we suggest two alternatives; increasing the number of training samples and changing the detection algorithm to a pixel-based approach such as semantic segmentation [31, 47]. To further improve the detection, we could also use contextual clues combined with feature detection. As an example, on the first pass for water point detection some locations were missed (such as Fig. 12g), results may have improved if an area proximity context scan had also been included, such as proximate buckets which had a high F-Score (Fig. 6) and mAP (Fig. 7) score. A cluster of these images, in combination with the water point prediction algorithm, might improve results. We found that just searching for clusters of buckets would not necessarily result in water points, but there is synergy in their combination. Again, this might need additional local training to determine appropriate contextualization (such as water container type).

It is hard to determine exactly how much additional local training would be required for the translation of these models to other countries. We suspect that certain features, like tires and drains are universal and can be successfully labelled from SV even without training, though as mentioned local configurations or contextualization would still improve results. Water points, toilets, street vendors and even the type of discarded trash might require additional training.

Next steps include determining the geographic variation in local training need by applying the results from this paper to other countries. This is vital because the type of SV data used in this paper is still not commonly available, though the technology is relatively inexpensive (approximately $150 per GPS enabled camera). While the authors have utilized SV in over twenty different countries, we acknowledge that more widespread adoption beyond the project team is still slow, though new groups do continually contact the authors for advice on implementation. The use of cell phone video collection in association with a GPS App in theory offers a more widespread utilization. However, the biggest impediment to growth in this method, is exactly what has been addressed in this paper, how to leverage data collected. What we have shown here is that these data can be turned into environmental health risk images when models are trained with the inclusion of local nuance. Next we will begin to merge image recognition with the associated coordinates simultaneously recorded within each frame of the SV so that these health risks can be automatically mapped once identified. Not only will this prove to be an important step forward in spatially supporting public health and epidemiological work in even the most challenging environments, but we believe this is the missing piece in making the SV method more widely utilized.

## Conclusion

Improving global health through hardware and software developments should be a research imperative. For the most challenging environments, a lack of data and logistical resources make disease prevention particularly difficult. SV data collection offers an exciting option for “mapping at the scale of intervention”. In this paper we have evolved this method further by showing how machine learning can be used to identify features typically associated with health risks from these videos. We have investigated how different environmental features vary in terms of model prediction, and how changes in the frequency of image selection, the type of object being classified, and even the image quality can vary results. We conclude that an SV—machine learning method is viable, and that in future, once these labeled video frames can be reattached to their associated GPS coordinates, then the prospect of an automatic mapping of dynamic challenging environments is an achievable goal.

## Data Availability

Not applicable.

## Footnotes

1 The selected neighborhoods included A in 2017 (two different cameras on the same route), 2018, and 2019; B in 2019; C in 2017; D in 2018 and 2019, and E in 2018.

2 For each of the categories, separate training tasks were initiated, being executed on a GPU-enabled Intel Core i7-9700 K CPU with 64 GB RAM. We have utilized NVIDIA GeForce GTX 1070 with 8 GB RAM for running Tensorflow optimized for GPU. For all the training tasks various quality parameters such as precision, recall, mean average precision (mAP), and F-score were captured.
